# Supplementary material for: Resistant and Susceptible Pinus thunbergii ParL. Show Highly Divergent Patterns of Differentially Expressed Genes during the Process of Infection by Bursaphelenchus xylophilus
Source: Int J Mol Sci. 2023 Sep 21;24(18):14376. doi: 10.3390/ijms241814376 (PMC10531596; doi:10.3390/ijms241814376)
Supplement: Supplementary file 1 [file ijms-24-14376-s001.zip › ijms-2583652-supplementary.pdf]

Table S1. Quality analysis of RNA and quantification of its purity and concentration.

RNA Gum Map No.	Sample names	Contents (ng/μl)	Total RNA (μg)	OD260/280	OD260/230	RIN
1	ph_1d_1	102.9	3.09	2.14	1.06	9.4
2	ph_1d_2	234.4	7.03	2.12	1.05	9.2
3	ph_1d_3	171.6	5.15	2.13	2.06	9.9
4	ph_3d_1	194.8	5.84	2.09	1.52	10
5	ph_3d_2	177.7	5.33	2.14	1.09	9.3
6	ph_3d_3	152.6	4.58	2.1	1.37	10
7	ph_7d_1	203.3	6.1	2.06	1.31	8.9
8	ph_7d_2	253.7	7.61	2.1	1.06	9.5
9	ph_7d_3	158.4	4.75	2.09	1.48	9.7
10	kh_1d_1	275.2	8.26	2.11	1.1	8.9
11	kh_1d_2	194.1	5.82	2.12	1	9.1
12	kh_1d_3	290.6	8.72	2.09	1.91	8.3
13	kh_3d_1	291.9	8.76	2.14	1.12	9.7
14	kh_3d_2	260.3	7.81	2.1	1.37	8.8
15	kh_3d_3	195.1	5.85	2.13	1.06	9.4
16	kh_7d_1	140.8	4.22	2.08	1.13	9.1
17	kh_7d_2	132.7	3.98	2.07	1.02	7.9
18	kh_7d_3	204.4	6.13	2.1	1.94	9.3
19	kh_14_1	90.7	2.37	2.24	0.17	9.7
20	kh_14_2	189.3	5.68	2.14	1.03	9.5
21	kh_14_3	217.3	7.61	2.12	1.58	9.3

Table S2. Primer pairs used in this study. Elongation factor 1-alpha was used as the internal control.

Gene ID	Primers	
Hypothetical protein	F: TGGAGTGACCTCGTAGAA	R: ATGTGACTTGAATGAAGACTT
Peroxidase	F: GAAGAGATGGAATGAGAT	R: CATTGAGAACAACAAGAT
Extracellular calcium sensing receptor	F: TTACCTATGCTTCTATCCA	R: GTATGTATCCTCCACCTT
Putative truncated TIR-NBS-LRR protein	F: ACGGTTATTCTGTGTTCT	R: CGAAGACTGTGGTAGATAT
Putative TIR/NBS/LRR disease resistance protein, partial	F: CTATACCTAATTGCTGCTCCAA	R: ACATATCATCGCCGTTCAC
Uncharacterized protein LOC110613552	F: TAGTTATGCCAATCTGATATG	R: ATCCTCTTCACCATCTTC
Glycerophosphodiester phosphodiesterase GDPDL7-like	F: TTACAAGCACCTACAAGT	R: GTCCACGAACTGAAGATA
Elongation factor-1 alpha	F: AGATTGGAAATGGTTATGCCCCTGT	R: CCAGAACGCCTGTCAACCTTGGT

Table S3 The expression of DEGs in susceptible and resistant *P. thunbergii*.

Host of PWN	gene name	gene id	gene set	FC	log2FC	*P* value
Susceptible *P. thunbergii*	SUM2	TRINITY_DN61527_c0_g1	ph_1d_vs_ph_3d	3.71000	1.89000	0.01864
	PR1	TRINITY_DN6837_c0_g1	ph_1d_vs_ph_3d	20.04000	4.36000	0.03495
	CAT1	TRINITY_DN56912_c1_g1	ph_1d_vs_ph_3d	280.87000	8.14000	0.03101
	PLR	TRINITY_DN57829_c0_g1	ph_1d_vs_ph_3d	0.05643	-4.14715	0.00000
PWN-resistant *P. thunbergii*	SUM2	TRINITY_DN61527_c0_g1	kh_1d_vs_kh_3d	3.34000	1.73859	0.03761
		TRINITY_DN56888_c0_g1	kh_1d_vs_kh_3d	2.90978	1.54091	0.02758
	PR1	TRINITY_DN21485_c0_g1	kh_1d_vs_kh_3d	18.05718	4.17450	0.00035
		TRINITY_DN6126_c0_g2	kh_1d_vs_kh_3d	46.59934	5.54224	0.00000
	CHIB	TRINITY_DN5952_c0_g1	kh_1d_vs_kh_3d	2.33988	1.22643	0.00898
		TRINITY_DN20491_c1_g1	kh_1d_vs_kh_3d	4.35911	2.12403	0.03017
		TRINITY_DN20491_c0_g1	kh_1d_vs_kh_3d	4.07870	2.02811	0.01869
	NDPK2	TRINITY_DN10975_c0_g1	kh_1d_vs_kh_3d	0.43377	-1.20499	0.02007
	PYL	TRINITY_DN15307_c0_g1	kh_1d_vs_kh_3d	0.17776	-2.49200	0.00147
		TRINITY_DN3450_c1_g1	kh_1d_vs_kh_3d	0.17031	-2.55377	0.00011
		TRINITY_DN3450_c0_g2	kh_1d_vs_kh_3d	0.29782	-1.74749	0.00000
		TRINITY_DN4825_c1_g1	kh_1d_vs_kh_3d	0.23541	-2.08677	0.00481
		TRINITY_DN4825_c0_g3	kh_1d_vs_kh_3d	0.09542	-3.38961	0.00003
		TRINITY_DN4825_c0_g1	kh_1d_vs_kh_3d	0.10069	-3.31200	0.00012
		TRINITY_DN57829_c0_g1	kh_3d_vs_kh7d	0.20872	-2.26031	0.00290
		TRINITY_DN20595_c0_g1	kh_3d_vs_kh7d	0.03937	-4.66671	0.00017
	PLR	TRINITY_DN18685_c0_g1	kh_3d_vs_kh7d	0.25122	-1.99293	0.01533

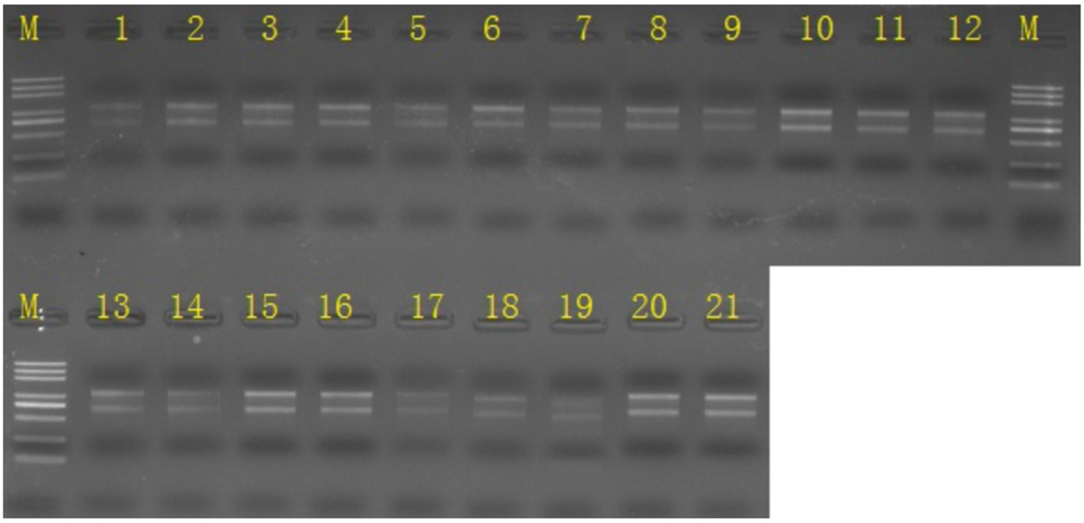

Figure. S1 The electropherograms presenting the RNA bands in agarose gels for twenty-one samples.

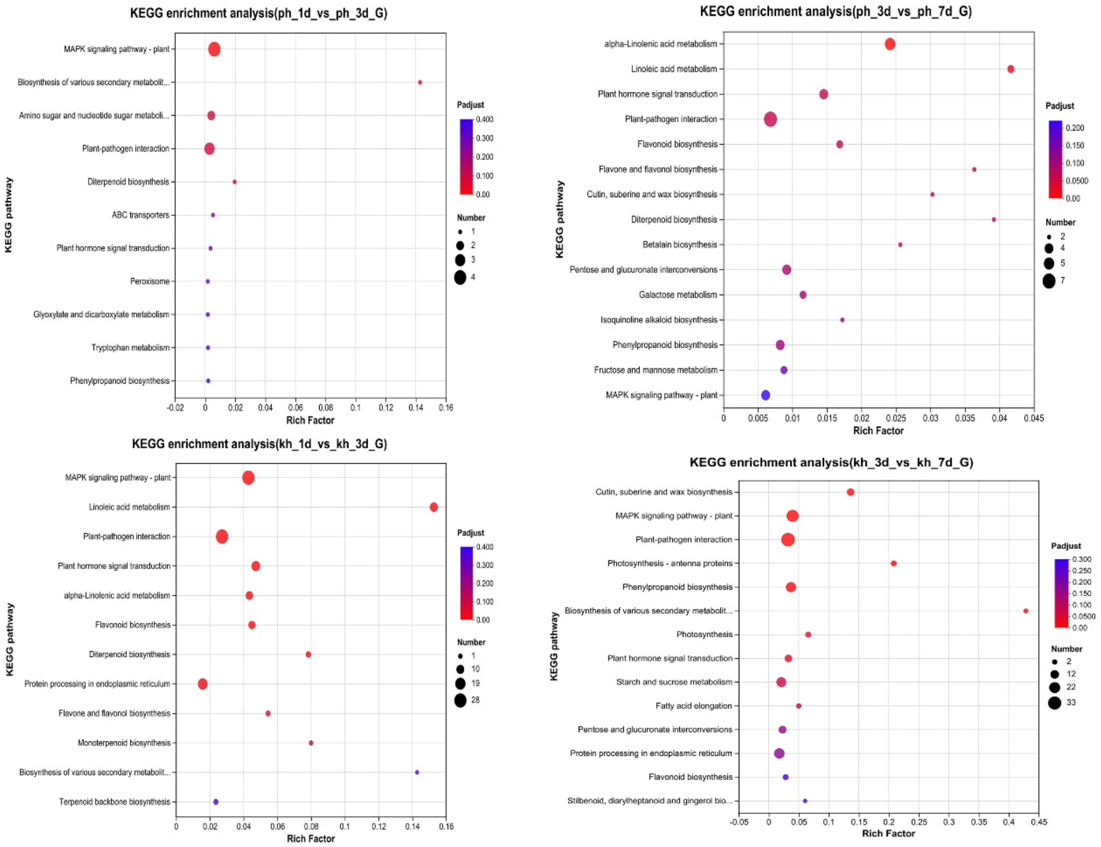

Figure. S2 the KEGG analysis of DEGs in susceptible and PWN-resistant *P. thunbergii*
